# Changes in whistle parameters of two common bottlenose dolphin ecotypes as a result of the physical presence of the research vessel

**DOI:** 10.7717/peerj.14074

**Published:** 2022-10-07

**Authors:** Simone Antichi, Jorge Urbán R., Sergio Martínez-Aguilar, Lorena Viloria-Gómora

**Affiliations:** Departamento de Ciencias Marinas y Costeras, Universidad Autónoma de Baja California Sur, La Paz, Baja California Sur, Mexico

**Keywords:** *Tursiops truncatus*, Gulf of California, Vocalization, Disturbance, Human impact, Cetaceans, Marine conservation

## Abstract

In the presence of vessels, dolphins have been found to change their habitat, behavior, group composition and whistle repertoire. The modification of the whistle parameters is generally considered to be a response to the engine noise. Little is known about the impact of the physical presence of vessels on dolphin acoustics. Whistle parameters of the coastal and oceanic ecotypes of common bottlenose dolphins in La Paz Bay, Mexico, were measured after the approach of the research vessel and its engine shutdown. Recordings of 10 min were made immediately after turning off the engine. For analysis, these recordings were divided from minute 0 to minute 5, and from minute 5:01 to minute 10. The whistles of the oceanic ecotype showed higher maximum, minimum and peak frequency in the second time interval compared to the first one. The whistle rate decreased in the second time interval. The whistles of the coastal ecotype showed no difference between the two time intervals. The physical presence of the research vessel could have induced a change in the whistle parameters of the oceanic dolphins until habituation to the vessel disturbance. The oceanic ecotype could increase the whistle rate and decrease the whistle frequencies to maintain acoustic contact more frequently and for longer distances. The coastal ecotype, showing no significant changes in the whistle parameters, could be more habituated to the presence of vessels and display a higher tolerance.

## Introduction

Recently, the development and diversification of human activities, especially in coastal waters, have increased the exposure of dolphins to a variety of anthropogenic disturbances (Sini et al., 2005). Industrial and recreational vessel traffic is one of the main threats and is predicted to rise (Erbe et al., 2019; McCarthy, 2004; McWhinnie et al., 2017; O’Connor et al., 2009). In the presence of vessels, dolphins have shown changes in habitat use (Lusseau, 2005; McCarthy, 2004), group composition and cohesion (Bejder et al., 2006; Miller, Solangi & Kuczaj, 2008). Dolphins can also interrupt foraging, resting or socializing activities in the presence of intense vessel traffic (Miller, Solangi & Kuczaj, 2008; Papale et al., 2015; Pirotta et al., 2015). Moreover, the engine noise of the vessels could provoke a change in the dolphin whistle parameters (Erbe et al., 2019; Jensen et al., 2009).

Dolphins use sounds for many aspects of their lives, such as navigation, communication and feeding (Gordon & Tyack, 2002). Bottlenose dolphins (*Tursiops truncatus*) rely on whistles for intra-specific communication, such as group cohesion, coordinated foraging and mother-calf interactions (Heiler et al., 2016; Janik & Sayigh, 2013; King & Janik, 2013). Whistles are omnidirectional narrowband sounds within a frequency range of 1–35 kHz (May-Collado & Wartzok, 2008; Richardson Jr et al., 2013). Each individual also produces a learned distinctive whistle with a stereotyped contour, interpreted as an acoustic signature, used to express identity information (Caldwell & Caldwell, 1965; Janik & Sayigh, 2013). Dolphins can react to vessel noise by adjusting duration, contour complexity, amplitude or frequencies of whistles to facilitate the transmission of their signals and to avoid acoustic masking (Fouda et al., 2018; Heiler et al., 2016; Kragh et al., 2019; May-Collado & Wartzok, 2008; Perez-Ortega et al., 2021; Rako et al., 2013). Furthermore, dolphins can increase (Buckstaff, 2004; Scarpaci et al., 2000) or decrease (Luís, Couchinho & dos Santos, 2014) whistle emission rate. In addition to the engine noise, the physical presence (Pirotta et al., 2015), the type (La Manna et al., 2013; Perez-Ortega et al., 2021) or the approach of the vessel during a sighting can have an impact on dolphins (Erbe et al., 2019). However, it is difficult to distinguish the contribution of each factor (Ellison et al., 2012). Most studies focus on the impact of the engine noise on the whistle parameters but there is little information about the impact of the vessel itself, with the engine off.

The common bottlenose dolphin is one of the most widely distributed dolphins in the world (Wells, Natoli & Braulik, 2019). Coastal and oceanic ecotypes of this species have been described in many regions, including the Gulf of California (Segura et al., 2006). The two ecotypes differ in habitat distribution, social structure, behavior (Bearzi, Saylan & Hwang, 2009; Viloria-Gómora & Medrano-González, 2015), phenotype (Gao, Zhou & Wang, 1995), diet (Díaz-Gamboa, 2003), genotype (Lowther-Thieleking et al., 2015; Segura et al., 2006), group size (Salinas Zacarías, 2005) and whistle repertoire (Hoffmann et al., 2012; Peters, 2018). Because of occupying two distinct habitats, the ecotypes are exposed to a different variety of threats, to which they could show different resistance (Wells, Natoli & Braulik, 2019). The constant interaction between the coastal ecotype and boats could make the dolphins familiar to the physical presence of vessels (Acevedo, 1991).

The present study focuses on the coastal and oceanic ecotypes of bottlenose dolphins found in La Paz Bay, in the Gulf of California. The investigation compares the whistle parameters of the two ecotypes in the first 5 min immediately after the approach of the research vessel and its engine shutdown, and the 5 min after this first period. Here, we hypothesize that the physical presence of the research vessel, even with the engine off, provokes a change in the whistle parameters of the dolphins. Furthermore, the reaction to the physical presence of the research vessel could be different between ecotypes given the difference of their habitat and the eventual different tolerances to the anthropogenic impact.

## Materials & Methods

**Figure 1:**
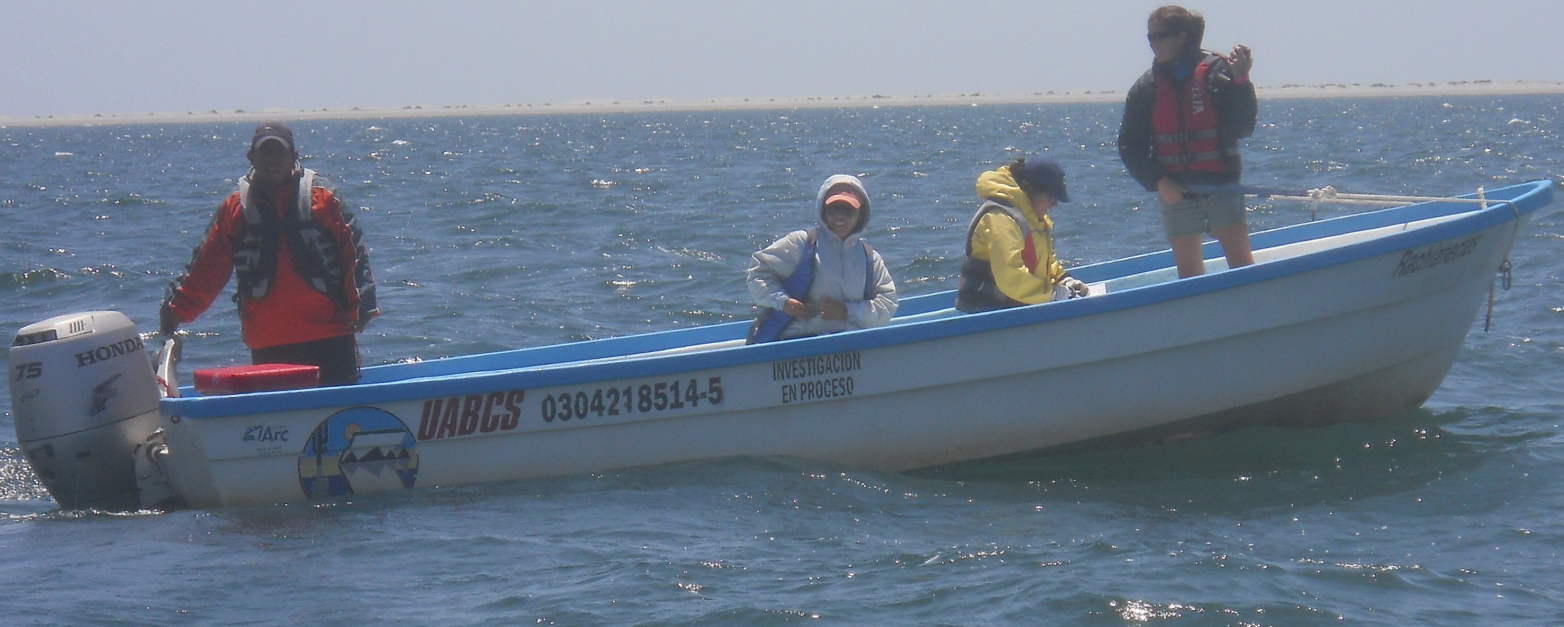
Research vessel used in the study. Photo credit: Hiram Rosales Nanduca.

The study area was La Paz Bay, located in the Baja California peninsula, Mexico, in the south-western part of the Gulf of California. Surveys were conducted with a 7.3 m research vessel (Fig. 1), between October 2020 and September 2021, only under favorable weather conditions (Beaufort scale ≤ 2). Observations were conducted through continuous scanning by naked eye and with binoculars (Mann, 1999).

The type (fishing panga; touristic panga; passenger panga; ferry; yacht; sailing boat; cargo; jet ski) and location of moving vessels, calculated using the distance (meters from the research vessel, estimated by naked eye) and absolute bearing (magnetic bearing, measured with a compass), were recorded as soon as vessels were visible. Panga is a type of small-sized (between 5 and 10 m) outboard-powered boat common in Mexico.

When a dolphin sighting occurred, the research vessel tracked parallel to the course of moving animals, approaching slightly to the rear of the group in a slow and continuous maneuver. At a maximum distance of 50 m from the dolphins the engine was turned off, and the hydrophone carefully deployed. The acoustic equipment was always prepared before the approach of the research vessel in order to start the recording immediately after the engine shutdown. Acoustic data were collected using a Reson TC4013.1 hydrophone (sensitivity −211 dB ± 3 dB re 1 V/µPa, frequency response 1 Hz to 170 kHz, omnidirectional) connected through a Reson VP2000 Voltage Preamplifier EC6081 (50 dB gain, 500 Hz high-pass filter, 50 kHz low-pass filter) to a Marantz PMD661 recorder (sampling rate 96 kHz, 24 bits resolution).

For each recording session ecotype and group size were registered, as well as behavior and group composition. Coastal and oceanic ecotypes were visually distinguished based on their differences in coloration and body size (Díaz-Gamboa, 2003; Salinas Zacarías, 2005; Segura et al., 2006). No mixed groups were encountered. Due to the difficulty to determine which individuals were being recorded, all visible dolphins were counted and considered as a single ‘acoustic’ group. The predominant behavior of the group (displayed by more than 50% of the dolphins) was recorded through notes using continuous scan sampling method (Altmann, 1974; Mann, 1999). The behavior was categorized into five behavioral state categories based on ethograms (Baker et al., 2017; Bearzi, 1994; Heiler et al., 2016) (Data S6). All behaviors were mutually exclusive. Group composition was defined as single (single individual), tight (each dolphin in the group was less than 1 body length from each other), loose (at least one dolphin was 1–5 body lengths from the others), dispersed (at least one dolphin was over 5 body lengths from the others), subgroups (tight groups were dispersed from each other), and patchy (large groups of dolphins were irregularly spread over an area) (Beddia, 2007). The recorded dolphin groups showed no behavioral transitions or changes in group composition at the moment of the research vessel approach. Thus, data on behavior and group composition were excluded from the analysis. All field experiments were conducted under a Scientific Research Permit issued by the Secretariat of Environment and Natural Resources of Mexico (SEMARNAT) (Permit SGPA/DGVS/00657/21). The study was entirely observational, and no ethical permit was required by the competent bodies. All procedures performed followed the “Guidelines for the treatment of marine mammals in field research” supported by the Society for Marine Mammalogy (Gales et al., 2009). The same trained researcher collected the observational data throughout all the study for consistency purposes.

**Figure 2:**
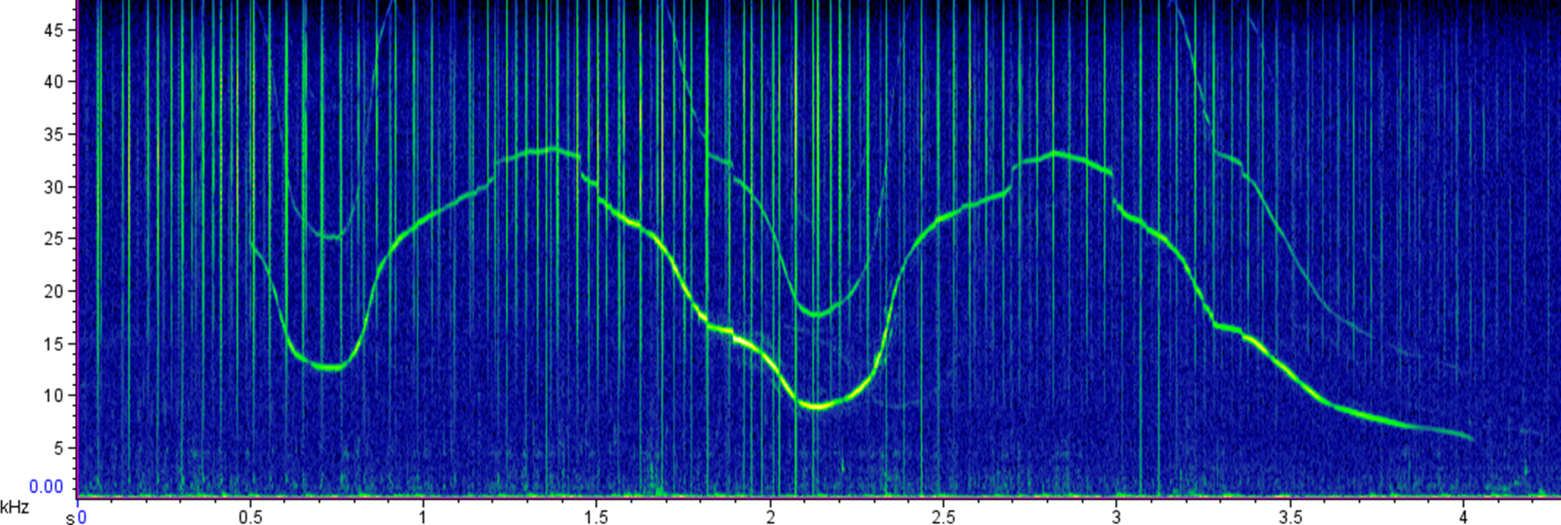
Sample spectrogram representing a common bottlenose dolphin whistle. Spectrogram: 512 points fast Fourier transform [FFT], Hann window, 50% overlap.

Acoustic recordings were first inspected in the spectrogram view of Raven Pro (version 1.5 Cornell University, Laboratory of Ornithology, New York) in the time-frequency domain (512 points fast Fourier transform (FFT), Hann window, 50% overlap). Non-overlapping whistles with the complete sound clearly visible in the spectrogram were selected (Fig. 2). The whistles were added to Luscinia software (version 2.16.10.29.01) (Lachlan, 2007). The spectrogram was set at 10 ms frame length, 5 ms time step, 48 kHz maximum frequency, 1,024 spectrograph point, Hann window, 50% overlap. The fundamental frequency contours were manually traced and the standard parameters: duration, starting frequency, ending frequency, minimum frequency, maximum frequency, frequency range and peak frequency (the frequency at which the maximum amplitude occurs in the whistle) were extracted (May-Collado & Wartzok, 2008; Perez-Ortega et al., 2021).

A total of 25 daily surveys was conducted in an effort of 179 h and 12 min. Common bottlenose dolphins were recorded 40 times and 1,208 whistles were detected in 1,091 min of recordings. To assess the impact of the research vessel, audio records with no other vessel audible or visible by naked eye were considered for the final analysis. To have the highest number of samples with a consistent duration, 10-minute samples of recordings were considered. Thus, from the full dataset, a selection of 347 whistles (coastal, *n* = 164; oceanic, *n* = 183) from 12 sightings (coastal, *n* = 8; oceanic, *n* = 4) recorded in absence of other vessels was analyzed (Table 1).

**Table 1:** Whistle selection process for the data analysis.

	**Full dataset**	**Analyzed dataset**
**Coastal ecotype**		
No. groups recorded	27	8
No. recordings	86	13
Recording duration (min)	668	130
No. whistles	675	164
**Oceanic ecotype**		
No. groups recorded	13	4
No. recordings	45	12
Recording duration (min)	423	120
No. whistles	533	183

Whistles were sorted according to their initial time (time associated to their starting frequency), and each recording was arbitrarily divided into two time intervals: first (from minute 0 to minute 5 after engine shutdown) and second (from minute 5:01 to minute 10 after engine shutdown) (Table 2). Time intervals of 5 min were selected in order to compare samples of equal duration and to obtain a higher number of whistles to analyze per sample. For each time interval, whistle rate (total whistles/group size/recording time) was measured (Quick & Janik, 2008). In addition, the stereotyped proportion was calculated as the ratio between the stereotyped whistles and the total whistles. Two or more whistles were defined as stereotyped when the signals had identical time-frequency contours visually matched by a trained observer (Papale et al., 2021). The normality and homoscedasticity of the data were checked by significance tests (Shapiro–Wilk and Levene’s test, respectively). As the assumption of normality was not applied for all the data, non-parametric tests were used. Mann–Whitney U-tests were performed to compare whistle parameters, rate and stereotyped proportion between the two time intervals. For the comparison of whistle parameters, to avoid the pseudo replication of stereotyped whistles, each stereotype was considered only once, and a final selection of 265 whistles (coastal, *n* = 123; oceanic, *n* = 142) were analyzed. Statistical analyses were performed in R software (version 4.0.5) with the RStudio interface (version 1.4.1106) using “stats” and “car” (Fox & Weisberg, 2019) packages.

**Table 2:** Analyzed whistles of the groups encountered.

**Ecotype**	**No. groups**	**Group size (min-max)**	**Group size (mean ± SD)**	**No. of whistles first time interval**	**No. of whistles second time interval**
Coastal	8	10–50	23 ± 15	81	83
Oceanic	4	30–150	68 ± 56	127	56

## Results

**Figure 3:**
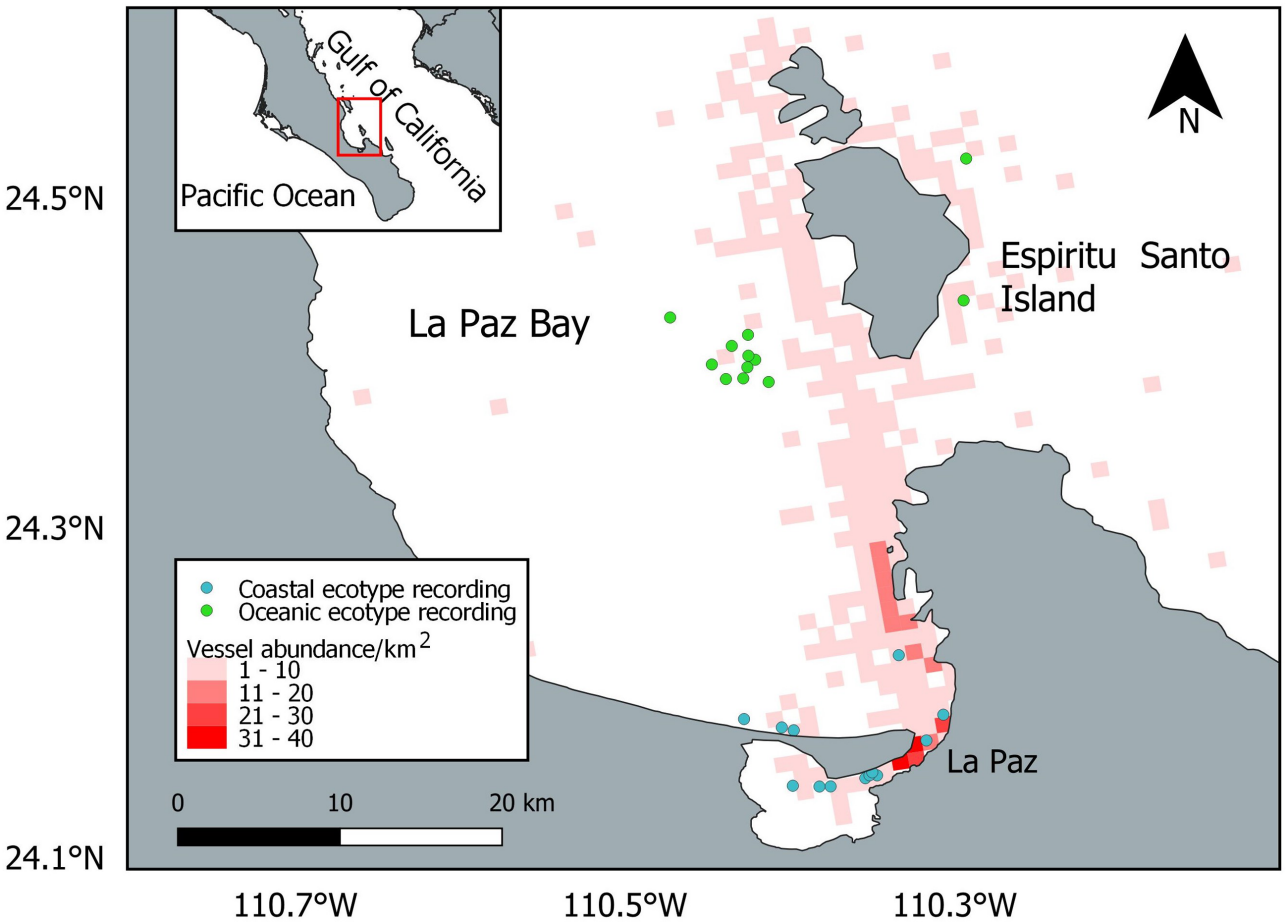
Location of the bottlenose dolphin recordings considered in the final analysis and vessel abundance. The basemap shapefile of Mexico is provided by Comisión Nacional para el Conocimiento y Uso de la Biodiversidad (CONABIO).

A total of 820 vessels were encountered during the entire study period, and the vessel abundance was overlapped with the dolphin locations used at the final analysis (Fig. 3). The most common type of vessel recorded was the touristic panga (*n* = 441; 54%), followed by the yacht (*n* = 161; 20%), the sailing boat (*n* = 96; 12%), the fishing panga (*n* = 57; 7%), the ferry (*n* = 34; 4%), the passenger panga (*n* = 15; 1%), the jet ski (*n* = 9; 1%), and the cargo (*n* = 7; 1%).

**Figure 4:**
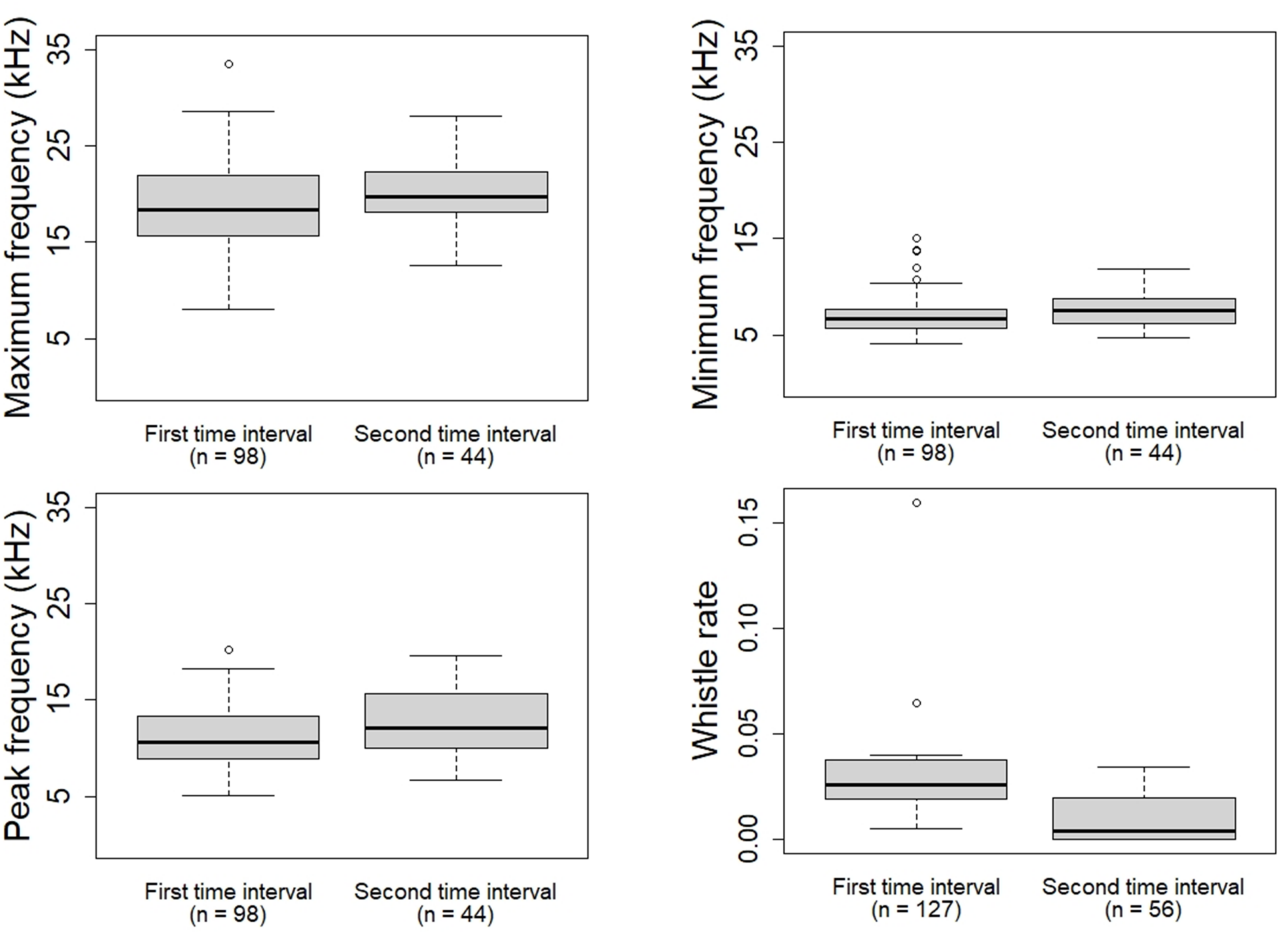
Whistle parameters and rate of the oceanic ecotype that showed significant difference between the two time intervals.

Descriptive statistics (mean, standard deviation, median) of the whistle parameters, rate and stereotyped proportion were calculated (Table 3; Table 4). During the first time interval the whistles of the oceanic ecotype showed higher whistle rate (*W* = 122.5, *p* = 0.003709), lower minimum frequency (*W* = 1,689.5, *p* = 0.0398), maximum frequency (*W* = 1,706.5, *p* = 0.04761) and peak frequency (*W* = 1,654, *p* = 0.02694) compared to the second time interval (Fig. 4). No significant differences were found in duration (*W* = 1,968, *p* = 0.4081), frequency range (*W* = 1,805, *p* = 0.1221), starting frequency (*W* = 2,329, *p* = 0.4467), ending frequency (*W* = 1,931, *p* = 0.322), and stereotyped proportion (*W* = 48.5, *p* = 0.6086). The whistles of the coastal ecotype showed no significant differences between the two time intervals: duration (*W* = 1,870, *p* = 0.9174), maximum frequency (*W* = 1,918, *p* = 0.8934), minimum frequency (*W* = 1, 862, *p* = 0.8854), frequency range (*W* = 1,954, *p* = 0.7519), starting frequency (*W* = 1,529.5, *p* = 0.06783), ending frequency (*W* = 2, 088, *p* = 0.3202), peak frequency (*W* = 1,802.5, *p* = 0.6562), whistle rate (*W* = 86, *p* = 0.959), and stereotyped proportion (*W* = 88.5, *p* = 0.3234).

**Table 3:** Descriptive statistics of the whistle parameters of the two bottlenose dolphin ecotypes.

	**First time interval (*n* = 61)**			**Second time interval (*n* = 62)**		
**Coastal**	**Mean**	**Sd**	**Median**	**Mean**	**Sd**	**Median**
Duration (s)	1.04	0.68	0.82	1.18	0.86	1.03
Maximum frequency (kHz)	15.21	3.07	15.13	15.25	3.65	14.54
Minimum frequency (kHz)	7.00	2.25	6.59	6.85	1.70	6.71
Frequency range (kHz)	8.21	3.27	7.64	8.40	4.10	7.38
Starting frequency (kHz)	9.70	3.92	9.00	11.11	4.26	10.65
Ending frequency (kHz)	9.42	3.15	8.79	8.72	2.38	8.25
Peak frequency (kHz)	9.80	2.63	9.56	9.93	2.67	9.69
	**First time interval** (***n = 98***)			**Second time interval (***n = 44***)**		
**Oceanic**	**Mean**	**Sd**	**Median**	**Mean**	**Sd**	**Median**
Duration (s)	1.14	0.53	1.10	1.29	0.72	1.21
Maximum frequency (kHz)^*^	18.69	4.54	18.39	20.05	3.44	19.65
Minimum frequency (kHz)^*^	6.97	2.09	6.70	7.51	1.80	7.48
Frequency range (kHz)	11.72	4.72	11.23	12.54	3.15	12.11
Starting frequency (kHz)	12.64	6.60	9.65	10.89	4.83	9.31
Ending frequency (kHz)	8.49	3.35	7.58	9.02	3.71	7.93
Peak frequency (kHz)^*^	11.21	3.30	10.56	12.81	3.82	12.06

**Notes.**

* Significantly different parameters between the two time intervals (Mann–Whitney *U*-tests, *p* < 0.05).

**Table 4:** Descriptive statistics of the whistle rate and stereotyped proportion of the two bottlenose dolphin ecotypes.

	**First time interval (*n* = 81)**			**Second time interval (*n* = 83)**		
**Coastal**	**Mean**	**Sd**	**Median**	**Mean**	**Sd**	**Median**
Whistle rate	0.06	0.05	0.05	0.07	0.10	0.04
Stereotyped proportion	0.31	0.35	0.16	0.17	0.24	0
	**First time interval** (***n = 127***)			**Second time interval** (***n = 56***)		
**Oceanic**	**Mean**	**Sd**	**Median**	**Mean**	**Sd**	**Median**
Whistle rate^*^	0.04	0.04	0.03	0.01	0.01	0
Stereotyped proportion	0.38	0.27	0.38	0.31	0.32	0.33

**Notes.**

* Significantly different rates between the two time intervals (Mann–Whitney *U*-tests, *p* < 0.05).

## Discussion

The present study finds differences in the whistle parameters and rate of the oceanic ecotype between the two time intervals after the approach of the research vessel and its engine shutdown. Results suggested that the physical presence of the research vessel, even with the engine off, could have affected dolphins and consequently their whistle repertoires. Although the engine noise is considered the medium through which the dolphins could perceive the approaching risk (Frid & Dill, 2002), the physical presence of vessels also seems to play a role in disturbance (Pirotta et al., 2015). Pirotta et al. (2015) found that the vessels presence, and not the noise level, was associated with a short-term reduction in foraging vocalizations (buzzes) of common bottlenose dolphins. The vessels that directly interacted with the dolphins provoked the greatest reduction in buzz occurrence.

The oceanic ecotype, five minutes after the approach of the research vessel and its engine shutdown, produced whistles at higher frequencies and at a lower emission rate compared to the first time interval. It could be possible that, in the second time interval, the dolphins got habituated to the presence of the research vessel and they had modified the whistle parameters and rate. Common bottlenose dolphins have been seen to modify whistle contour during stressful situations. Esch et al. (2009) reported dolphins showing higher whistle rates during capture-release events compared to undisturbed conditions. This result is in accordance with the present study where the oceanic ecotype displayed a higher whistle rate in the first time interval compared to the second one. The increase of the whistle rate could reflect a higher motivation to maintain acoustic contact; while the lower-frequency whistles may allow the dolphins to cover longer distances and communicate with more individuals of the group. Alternatively, the presence of the research vessel could provoke excitement and curiosity of the dolphins, which could tend to increase the communication between each other (Norris et al., 1994; Wells, 1980). Despite the engine shutdown, it is possible that the dolphins could still have been affected by the noise previously emitted. Dolphins may have started modifying the whistle parameters and rate when the research vessel was moving, and continued for the first minutes after the engine shutdown. However no acoustic recordings were made with the engine on as it would have been difficult to discern whether the dolphins were reacting to the physical presence of the research vessel or to its engine noise. Furthermore, due to the engine noise of the research vessel, the selection of whistles with the complete sound clearly visible in the spectrogram for the analysis could have been difficult. Comparing the results with a control situation in absence of the research vessel could have helped to better understand the impact of its physical presence. However, acoustic data collection without research vessel was not feasible as it would have not allowed to confirm the presence/absence of vessels or the dolphin ecotype. Only passive acoustic monitoring together with land surveys could have created a control situation but it was not applicable because of the complexity of the study area (particularly the oceanic part not close enough to any land).

While the oceanic ecotype modified the whistle parameters, the coastal dolphins showed no variation. This result is in contrast with the hypothesis that the level of perceived risk may be higher for small groups of dolphins (Frid & Dill, 2002), as the coastal ecotype ones were compared to the oceanic in this study. Bottlenose dolphins already showed whistle variation between populations (La Manna et al., 2020; Luís et al., 2021; May-Collado & Wartzok, 2008; Papale et al., 2021) and ecotypes (Hoffmann et al., 2012; Peters, 2018; Rio et al., 2022), possibly due to geographical distance, group size and composition, behavioral activities, genetic isolation, morphology, and habitat acoustic characteristics. The peculiarity of the results found in the present study is the different reaction to the physical presence of the research vessel between the coastal and oceanic ecotypes. A possible explanation of this difference could be the different habitat they occupy. The coastal ecotype was encountered close to La Paz, in an area with a higher abundance of vessels compared to the oceanic habitat (Fig. 3), and could thus be more habituated to the presence of vessels. The dolphins could result unaffected by vessel presence because they are constantly exposed to and become familiar with it (Acevedo, 1991; Gregory & Rowden, 2001). Stereotyped whistles may encode individual identity and could be used as contact calls (Caldwell, Caldwell & Tyack, 1990), between mother and calf pairs (Smolker, Mann & Smuts, 1993) or to facilitate group cohesion (Janik, 2009). In this study neither ecotype changed the stereotyped whistle proportion during the recording time. The response of dolphins to vessel presence could depend on the initial behavior (May-Collado & Quiñones Lebrón, 2014), presence of calves (Guerra et al., 2014; Heiler et al., 2016) and group size (Pellegrini et al., 2021). More research is needed to investigate the reaction to the vessel presence in different social situations.

Despite the precautions taken in the vessel maneuvers to minimize its possible impact, a change of the whistle parameters was recorded, highlighting the importance of including the research vessel as a factor in acoustic studies of dolphins. The touristic panga was the most encountered vessel, confirming that touristic activities take place regularly in La Paz Bay. However, no regulations for dolphin observations exist in the Mexican law and whale-watching activities are only regulated for baleen whales and sperm whales (DOF, 2011; Urbán & Viloria-Gómora, 2021). The research vessel used in this study is a panga vessel similar in size and engine to the touristic pangas present in La Paz Bay. Pangas, with no precautions in the approach of dolphins, could thus have greater impacts than the one demonstrated in this study, where approaching maneuvers were careful. Touristic activities mainly occur along the coastline, but touristic vessels may also cross offshore waters, overlapping with the habitat of the oceanic ecotype, especially on the way to Espiritu Santo Island (Fig. 3). Although sea lions, whale sharks and humpback whales are the main attractions in the bay, in case of a sighting, vessels may follow common bottlenose dolphins as well. Limiting the number of vessels and increasing the distance from the animals may help to reduce the disturbance during sightings (Jensen et al., 2009). Specific guidelines such as driving at a steady speed and parallel to the group of dolphins could be followed by the captains to hinder the impact (Jensen et al., 2009).

## Conclusions

This study indicates that the oceanic bottlenose dolphin ecotype modified the whistle parameters and rate 5 min after the approach of the research vessel and its engine shutdown. The oceanic ecotype may need to increase the acoustic contact inside the group immediately after the approach of the research vessel. The coastal dolphins, possibly being more habituated to vessel encounters, may not change their whistle parameters. More research is needed to better understand the different factors that could influence the reaction to the physical presence of the research vessel.

##  Supplemental Information

10.7717/peerj.14074/supp-1 Supplemental Information 1 Total effort of the study period

10.7717/peerj.14074/supp-2 Supplemental Information 2 Whistle parameters comparison

10.7717/peerj.14074/supp-3 Supplemental Information 3 Whistle rate and stereotyped proportion

10.7717/peerj.14074/supp-4 Supplemental Information 4 Vessel type and location

10.7717/peerj.14074/supp-5 Supplemental Information 5 Statistical analysis conducted in the study

10.7717/peerj.14074/supp-6 Supplemental Information 6 Common bottlenose dolphin ethogram

10.7717/peerj.14074/supp-7 Supplemental Information 7 Audio sample of a common bottlenose dolphin whistle

## Additional Information and Declarations

### Data Availability

The following information was supplied regarding data availability: The raw data is available in the Supplementary Files.
