# Supplementary material for: Changes in whistle parameters of two common bottlenose dolphin ecotypes as a result of the physical presence of the research vessel
Source: PeerJ. 2022 Oct 7;10:e14074. doi: 10.7717/peerj.14074 (PMC9549881; doi:10.7717/peerj.14074)
Supplement: Supplemental Information 6 [file peerj-10-14074-s006.docx]

| **Behavior** | **Description** |
| --- | --- |
| Feeding | The group is pursuing prey and feeding (often confirmed by visual observation of the prey). This behavior could be associated with deep diving, fast swims or porpoising. Presence of seabirds in the area could be an indicator of feeding behavior. |
| Traveling | The group is moving in a consistent direction with regular surfacing intervals. |
| Socializing | The individuals of the group are in almost constant physical contact with each other, often displaying surface behaviors (breach, leap etc.) and no steadily directional movement. |
| Milling | No net movement; individuals surfacing facing different directions; pod often changes direction; dive intervals vary. May indicate a transitory phase. |
| Resting | Slow, directed movement (slower than speed of vessel); no splashing; closely grouped or in subgroups; short, relatively constant, synchronous dive intervals. |
